# DDX39 promotes hepatocellular carcinoma growth and metastasis through activating Wnt/β-catenin pathway

**DOI:** 10.1038/s41419-018-0591-0

**Published:** 2018-06-04

**Authors:** Tong Zhang, Zhenjiang Ma, Lijuan Liu, Jian Sun, Hui Tang, Bing Zhang, Ying Zou, Heping Li

**Affiliations:** 1 Department of Hepatic Surgery, Liver Transplant Center, the Third Affiliated Hospital of Sun Yat-sen University, Guangzhou, 510630 China; 2 Liver Transplantation Center of Sun Yat-sen University, Guangzhou, 510630 China; 3 Organ Transplantation Institute of Guangdong Province, Guangzhou, 510630 China; 4 Department of Interventional Radiology, the First Affiliated Hospital of Sun Yat-sen University, Guangzhou, 510080 China; 5 Department of Cardiology, the First Affiliated Hospital, Sun Yat-Sen University, Guangzhou, 510080 China; 6 Department of Hepatobiliary and pancreatic Surgery, Sun Yat-Sen Memorial Hospital, Sun Yat-Sen University, Guangzhou, 510120 China; 7 Guangdong Provincial Key Laboratory of Malignant Tumor Epigenetics and Gene Regulation, Medical Research Center, Sun Yat-Sen Memorial Hospital, Sun Yat-Sen University, Guangzhou, 510120 China; 8 Department of Medical Imaging, the First Affiliated Hospital of Sun Yat-sen University, Guangzhou, 510080 China; 9 Department of Medical Oncology, the First Affiliated Hospital of Sun Yat-sen University, Guangzhou, 510080 China

## Abstract

Hepatocellular carcinoma (HCC) is the third leading cause of cancer related death worldwide; however, the molecular mechanisms regulating HCC progression remain largely unknown. In this study, we determined the role of DDX39 which a DEAD-box RNA helicase in HCC progression, and found DDX39 was upregulated in HCC tissues and cells, DDX39 expression was positive correlated with advanced clinical stage, survival analysis showed patients with high-DDX39 levels had poor outcome, it was an independent prognostic factor. Functional analysis revealed that DDX39 overexpression promoted HCC cell migration, invasion, growth, and metastasis, DDX39 knockdown inhibited HCC cell migration, invasion, growth, and metastasis. Mechanism analysis suggested DDX39 overexpression increased β-catenin expression in nucleus and increased Wnt/β-catenin pathway target genes levels, while DDX39 knockdown reduced this effect. Knockdown of Wnt/β-catenin pathway co-activators TCF4 and LEF1 in DDX39 overexpressing HCC cells inhibited Wnt/β-catenin pathway target genes. The invasion ability was also reduced, confirming DDX39 regulates HCC progression by activating Wnt/β-catenin pathway. In conclusion, we found DDX39 is a target and prognostic factor for HCC, and promotes HCC migration, invasion, growth, and metastasis by activating Wnt/β-catenin pathway.

## Introduction

DDX39 is a DEAD box RNA helicase which unwind double-stranded RNA in an ATP-dependent manner, it interacts with HCC-1, U2AF65, REF2-1, TRF2, ALY, CIP29, and FUS/TLS to regulate transcription, splicing, RNA export, ribosome biogenesis, telomere protection and translation^1–7^, DDX39 also regulates tumor progression. DDX39 is overexpressed in lung squamous cell cancer, and promotes cancer cell proliferation^8^. DDX39 is an independent prognostic biomarker for gastrointestinal stromal tumor, and associated with metastasis^9^. It inhibits invasion of bladder cancer and is an unfavorable prognostic factor for bladder cancer^10^. DDX39 is a putative biomarker of breast cancer and early-stage hepatocellular carcinoma^11,12^. DDX39 also upregulates in malignant pleural mesothelioma cells and pancreatic cancer cells acquired gemcitabine resistance^13,14^. Although DDX39 has been reported to regulate the progression of many tumors, but its role in tumor development and regulatory mechanisms has not been understood well. Here we analyzed the prognostic value of DDX39 in HCC, and studied the role of DDX39 in HCC migration, invasion, metastasis, and growth, we found DDX39 was an unfavorable prognosis for HCC patients, and promoted HCC metastasis and growth. Then we studied the regulatory mechanism of DDX39, and found DDX39 promoting HCC progression though activating Wnt/β-catenin pathway.

## Results

### DDX39 is an independent prognostic factor for HCC patients

To investigate the role of DDX39 in HCC progression, we first analyzed DDX39 expression in HCC tissues using data downloaded from GSE14520 which contains gene expression date of HCC, and found DDX39 was significantly upregulated in HCC tissues (Supplemental Fig. 1A). GSEA analysis suggested patients with high-DDX39 expression had short survival time, patients with low-DDX39 expression had long survival time (Supplemental Fig. 1B), suggesting DDX39 might be a prognostic factor for HCC patients. Oncomine database suggested DDX39 was upregulated in many tumors, including liver cancer (Supplemental Fig. 1C). These results suggested DDX39 might promote HCC progression.

**Fig. 1:**
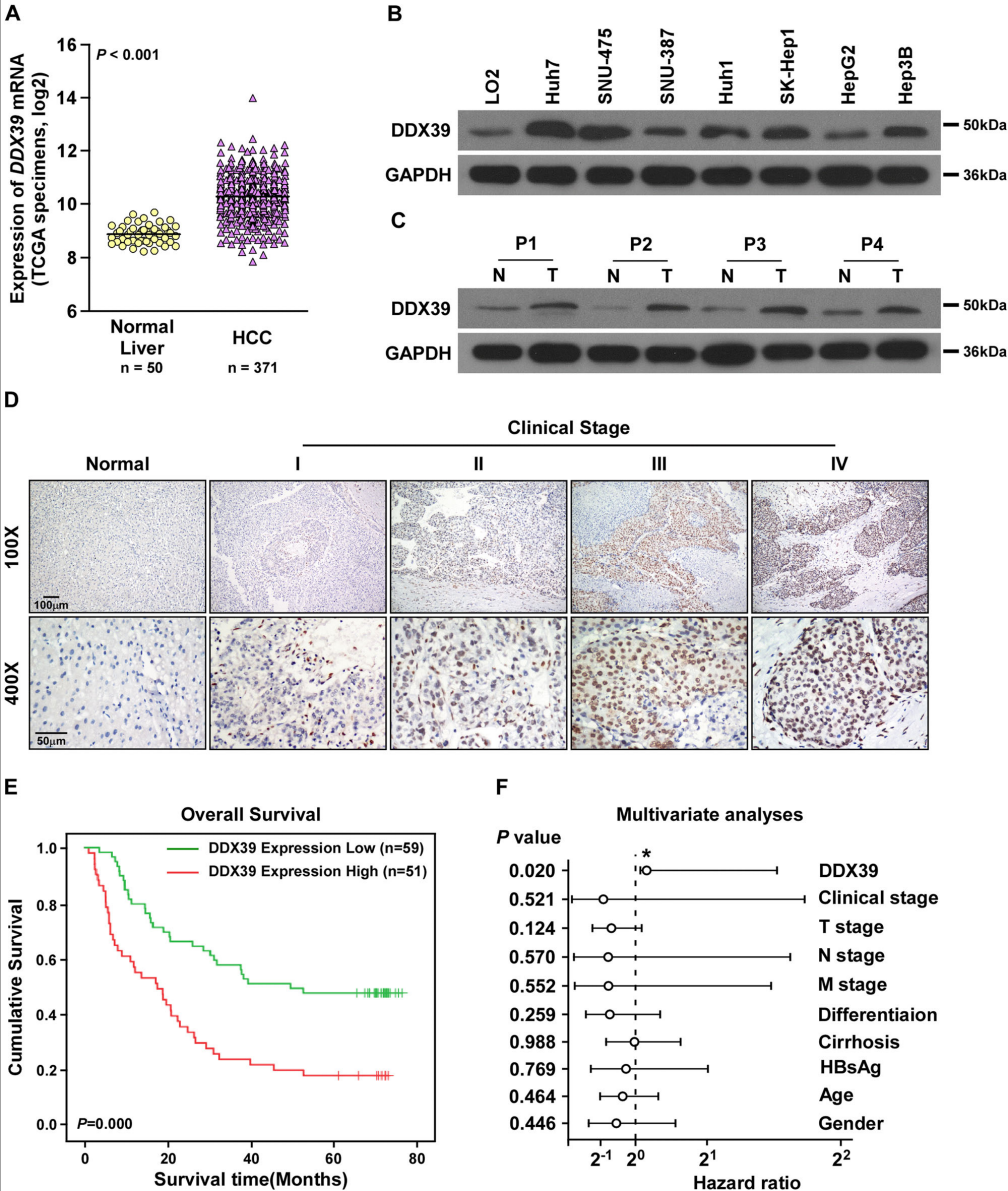
DDX39 is an unfavorable prognostic factor for HCC patients. **a** DDX39 was upregulated in HCC tissues compared to normal liver tissues, data were downloaded from TCGA dataset. **b** Western blot analyzed DDX39 levels in normal liver cell and HCC cells. GAPDH was used as the loading control. **c** Western blot analyzed DDX39 levels in four pairs of HCC tissues (T) and matched normal liver tissues (N). GAPDH was used as the loading control. **d** IHC assay for DDX39 expression in normal liver tissues and HCC tissues with stage I–IV, representative images of IHC assay were shown. **e** Kaplan–Meier analysis of survival of HCC patients, patients whose tumors with DDX39 overexpression were labeled in red, and whose tumors with DDX39 underexpression were labeled in green. **f** Multivariate analyses of the prognostic value of DDX39 levels and other clinicopathologic characteristics, such as clinical stage, T stage, N stage, M stage, differentiation, cirrhosis, HBsAg, age and gender. *p < 0.05

We used TCGA date further to study DDX39 expression in HCC, and found DDX39 was significantly upregulated in HCC tissues compared to normal liver (Fig. 1a). DDX39 was upregulated in HCC cells compared to the normal liver cell (Fig. 1b and Supplemental Fig. 2A). We used another four paired of HCC tissues and adjacent liver tissues to determine DDX39 expression, and found DDX39 was also upregulated in four HCC tissues compared to the adjacent normal liver tissues both in mRNA and protein levels (Fig. 1c and Supplemental Fig. 2B). We also determined DDX39 levels in normal liver tissues and HCC tissues (*n* = 110) using IHC, suggesting DDX39 was upregulated in HCC tissues, and patients with advanced HCC stage had higher DDX39 levels (Fig. 1d and Supplemental Fig. 2C). Survival cure analysis suggested patients with high-DDX39 levels had poor outcome, patients with low-DDX39 levels had good outcome (Fig. 1e). Multivariate analyses suggested DDX39 was an independent prognostic factor for HCC patients (Fig. 1f). These findings suggested DDX39 was upregulated in HCC cells and tissues, and was an unfavorable prognostic factor for HCC patients.

### DDX39 contributes to HCC migration, invasion, and metastasis

**Fig. 2:**
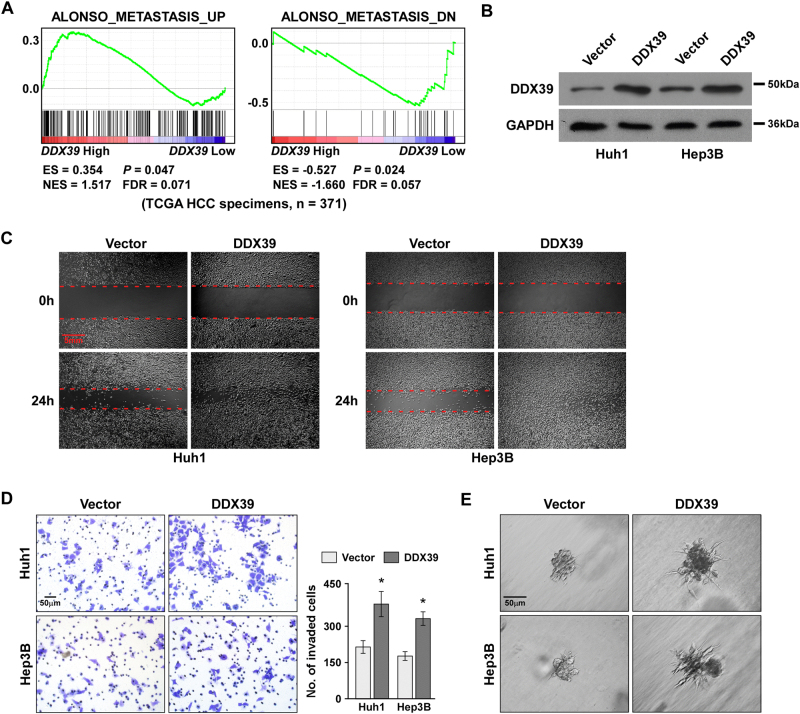
DDX39 overexpression promoted HCC cell migration and invasion. **a** GSEA plot suggested DDX39 expression positively correlated with metastasis associated gene signatures using TCGA data. **b** Western blot analysis of DDX39 levels after DDX39 overexpression in Huh1 and Hep3B. GAPDH was used as the loading control. **c** Wound healing assay of the effect of DDX39 overexpression on HCC cell migration. **d** Transwell assay of the effect of DDX39 overexpression on HCC cell invasion. **e** 3D spheroid invasion assay of the effect of DDX39 overexpression on HCC cell invasion. Data are shown as means ± SD. *p < 0.05

To analyze DDX39’s role in HCC development, GSEA analysis was performed to analyze the correlation between DDX39 levels and HCC metastasis, and suggested patients with high-DDX39 levels had high-metastatic ability, patients with low-DDX39 levels had low metastatic ability, DDX39 levels were positive correlation with tumor metastasis (Fig. 2a). Thus, we determined the role of DDX39 in HCC migration, invasion, and metastasis. DDX39 was overexpression in HCC cells Huh1 and Hep3B (Fig. 2b). Wound healing assay suggested DDX39 overexpression promoted HCC cell migration (Fig. 2c). Transwell assay suggested DDX39 overexpression significantly promoted HCC cell invasion (Fig. 2d). Three-dimensional spheroid invasion assay suggested DDX39 overexpression increased invasive projections emanating from cells compared to the empty vector control group (Fig. 2e). These findings suggested DDX39 overexpression promoted HCC cell migration and invasion.

**Fig. 3:**
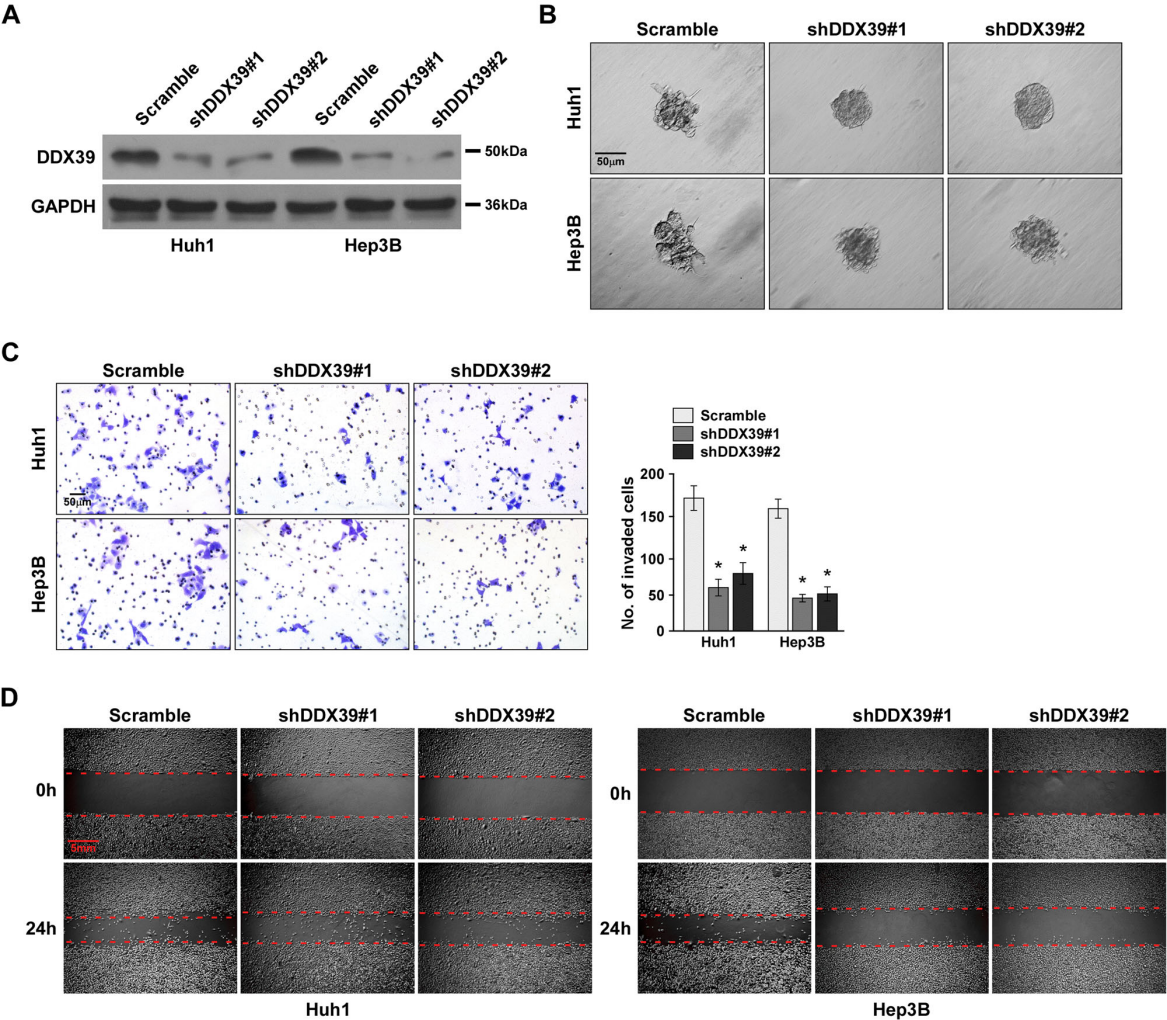
DDX39 knockdown inhibited HCC cell migration and invasion. **a** Western blot analysis of DDX39 levels after DDX39 knockdown in Huh1 and Hep3B. GAPDH was used as the loading control. **b** 3D spheroid invasion assay of the effect of DDX39 knockdown on HCC cell invasion. **c** Transwell assay of the effect of DDX39 knockdown on HCC cell invasion. **d** Wound healing assay of the effect of DDX39 knockdown on HCC cell migration. Data are shown as means ± SD. *p < 0.05

To confirm above findings, we knocked down DDX39 in the same HCC cells (Fig. 3a). 3D spheroid invasion assay suggested DDX39 knockdown reduced invasive projections emanating from cells compared to the scramble control group (Fig. 3b). Transwell assay suggested DDX39 knockdown significantly inhibited HCC cell invasion (Fig. 3c). Wound healing assay suggested DDX39 knockdown inhibited HCC cell migration (Fig. 3d). These findings suggested DDX39 knockdown suppressed HCC cell migration and invasion. Together, these findings suggested DDX39 contributed to HCC cell migration and invasion.

**Fig. 4:**
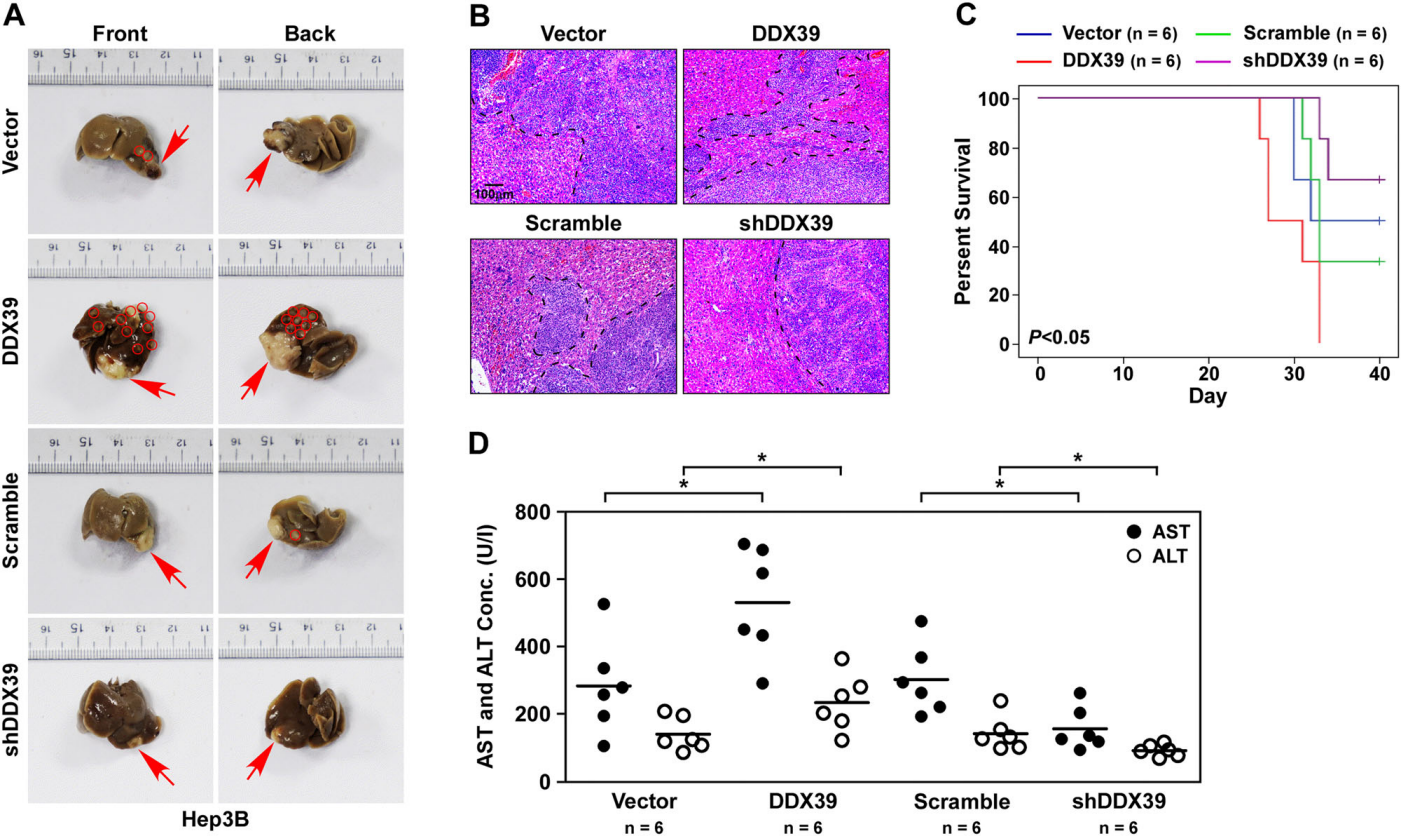
DDX39 contributes to HCC metastasis and growth. **a** Orthotopic transplantation tumor model analyzed the role of DDX39 on tumor growth and lung metastasis, representative images of tumors derived from Hep3B with DDX39 overexpression or knockdown in lung and liver. **b** HE staining of tumors derived from Hep3B with DDX39 overexpression or knockdown in lung. **c** Kaplan–Meier analysis of survival for mice transplanted with Hep3B with DDX39 overexpression or knockdown. **d** The activity of AST and ALT in blood of mice transplanted with Hep3B with DDX39 overexpression or knockdown. *p < 0.05

Orthotopic transplantation tumor model was used to determine whether DDX39 regulates distant metastasis and growth, and found DDX39 overexpression promoted HCC growth and lung metastasis, the volume of tumors with DDX39 overexpression was larger than empty vector group, DDX39 overexpression increased the number of tumor nodules in lung. DDX39 knockdown inhibited HCC growth and lung metastasis, the volume of tumors with DDX39 knockdown was smaller than the scramble control group, DDX39 knockdown reduced the number of tumor nodules in lung (Fig. 4a), suggesting DDX39 promoted HCC growth and metastasis. HE staining also showed DDX39 overexpression promoted lung metastasis of HCC, DDX39 knockdown inhibited lung metastasis of HCC (Fig. 4b). Survival cure analysis of mouse transplanted with HCC cell with DDX39 overexpression or knockdown showed DDX39 overexpression shorted the survival time of mouse, DDX39 knockdown increased the survival time of mouse (Fig. 4c). AST and ALT are important diagnostic markers for liver disease, DDX39 overexpression significantly increased AST and ALT concentrations, while DDX39 knockdown significantly decreased AST and ALT activity (Fig. 4d). Together, these findings suggested DDX39 promoted HCC growth and metastasis.

### DDX39 promotes HCC development through activating Wnt pathway

**Fig. 5:**
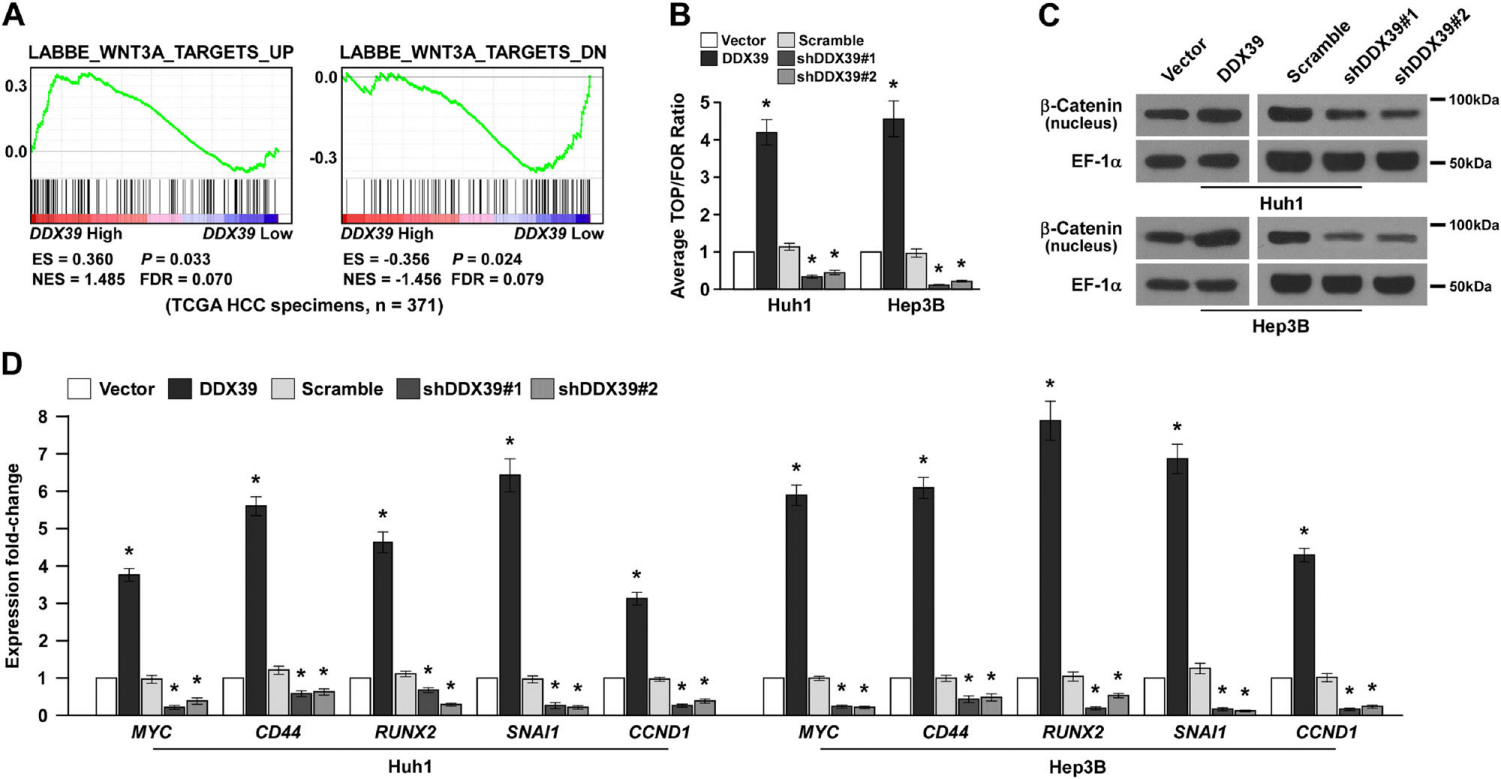
DDX39 activates Wnt/β-catenin pathway. **a** GSEA analysis of the correlation between DDX39 levels and Wnt3A target gene signatures using TCGA data. **b** TOP/FOP luciferase activity assay revealed DDX39 overexpression activated Wnt/β-catenin pathway. DDX39 knockdown inhibited Wnt/β-catenin pathway. **c** Western blot analysis of β-catenin levels in nucleus of HCC cells with DDX39 overexpression or knockdown, EF-1α was used as the loading control. **d** qRT-PCR analyzed the expression of Wnt/β-catenin target genes like MYC, CD44, RUNX2, SNAIL1, and CCND1 in HCC cells with DDX39 overexpression or knockdown. Data are shown as means ± SD. *p < 0.05

To determine the regulatory mechanism of DDX39 promoting HCC development, we used GSEA analysis to predict the pathways regulated by DDX39. High-DDX39 levels were positive corrected with Wnt3A targets levels (Fig. 5a), suggesting DDX39 might regulate Wnt pathway. TOP/FOP luciferase activity assay suggested DDX39 overexpression significantly activated Wnt/β-catenin pathway, DDX39 knockdown significantly inactivated Wnt/β-catenin pathway (Fig. 5b). β-catenin (also known as CTNNB1) translocated to nucleus is the marker for Wnt/β-catenin pathway activation, western blot assay suggested DDX39 overexpression increased β-catenin levels in nucleus, DDX39 knockdown inhibited β-catenin levels in nucleus, suggesting DDX39 activated Wnt/β-catenin pathway (Fig. 5c). We also determined the expression of target genes of Wnt/β-catenin pathway, such as MYC^15^, CD44^16^, RUNX2^17^, SNAI1^18^, and CCDN1 (Cyclin D1)^19^, DDX39 overexpression significantly promoted their expression, DDX39 knockdown significantly inhibited their expression (Fig. 5d). These findings suggested DDX39 could activate Wnt/β-catenin pathway.

**Fig. 6:**
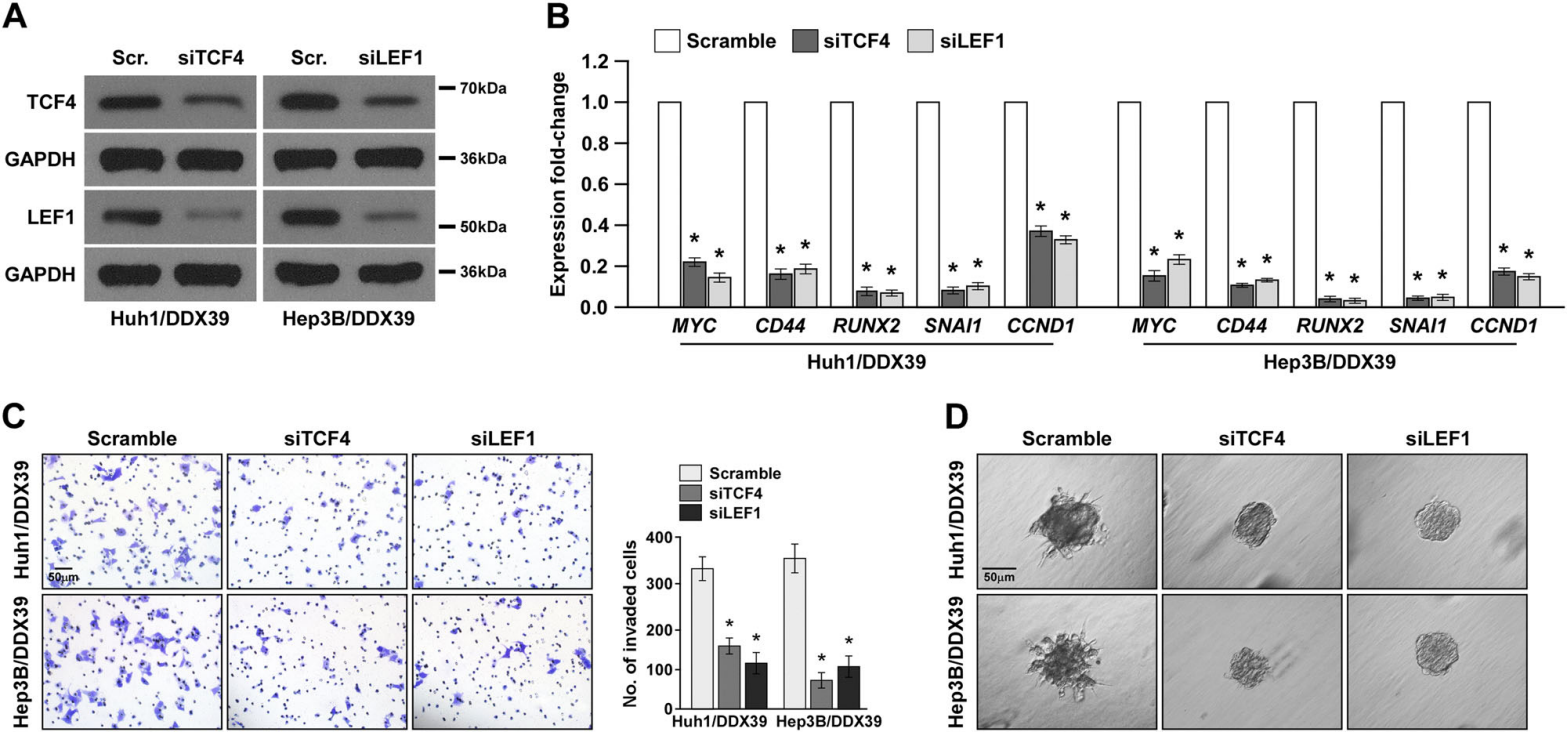
DDX39 promotes HCC progression through activating Wnt/β-catenin pathway. **a** Western blot assay of TCF4 and LEF1 expression in HCC cells overexpressing DDX39 transfected with siRNAs for TCF4 or LEF1. **b** qRT-PCR analyzed the expression of Wnt/β-catenin target genes like MYC, CD44, RUNX2, SNAIL1, and CCND1 in HCC cells overexpressing DDX39 transfected with siRNAs for TCF4 or LEF1. **c** Transwell assay of the effect of TCF4 or LEF1 knockdown in HCC cells overexpressing DDX39 on cell invasion. **d** 3D spheroid invasion assay of the effect of TCF4 or LEF1 knockdown in HCC cells overexpressing DDX39 on cell invasion. Data are shown as means ± SD. *p < 0.05

To confirm above results, we knocked down TCF4 and LEF1 in HCC cells with DDX39 overexpression to inactivate β-catenin pathway (Fig. 6a), TCF4 and LEF1 are the co-activators of Wnt/β-catenin pathway. Knockdown of TCF4 or LEF1 in HCC cells with DDX39 overexpression significantly inhibited MYC, CD44, RUNX2, SNAI1, and CCND1 expression (Fig. 6b). Transwell assay suggested knockdown of TCF4 or LEF1 significantly inhibited HCC cell invasion (Fig. 6c). 3D spheroid invasion assay suggested knockdown of TCF4 or LEF1 reduced invasive projections emanating from cells (Fig. 6d). TCF4 or LEF1 knockdown reversed the phenotypes caused by DDX39 overexpression, confirming DDX39 regulated HCC progression through activating Wnt/β-catenin pathway.

## Discussion

In this study, we studied the role of DDX39 in HCC prognosis and progression. DDX39 was upregulated in HCC tissues and cells, high-DDX39 levels positively correlated with advanced clinical stages, high-DDX39 levels were associated with poor outcome. DDX39 overexpression promoted HCC migration, invasion, metastasis, and growth, while DDX39 knockdown reduced these effects. Mechanism analysis suggested DDX39 promoted HCC progression by activating Wnt/β-catenin pathway.

Although, DDX39 has been shown to regulate several tumors progression, but they only performed in vitro assay, and the molecular mechanism also could not be carried out. Our research analyzed the DDX39’s role in HCC progression in vitro and in vivo, and found DDX39 promoted HCC growth and lung metastasis. We also studied the molecular mechanism, and found DDX39 could activate Wnt/β-catenin pathway.

Wnt/β-catenin pathway has been well-recognized and well-studied. Wnt ligands binding to its receptor initiate the Wnt signaling, β-catenin dissociates from the destruction complex composed of Axin, adenomatous polyposis coli (APC) and GSK3β, and translocates into nucleus. Then β-catenin binds to co-activators LEF and TCF to form transcriptional complex, and regulates the Wnt target genes expression^20,21^. Wnt/β-catenin pathway promotes HCC growth, metastasis and the self-renewal of HCC stem cells^22–26^. We found DDX39 promoted β-catenin accumulate in nucleus, and the expression of Wnt signaling target genes, suggesting DDX39 activated Wnt signaling. Knockdown of Wnt/β-catenin pathway coactivator TCF4 and LEF1 in HCC cells with DDX39 overexpressing inhibited HCC progression, confirming DDX39 promoted HCC progression by activating Wnt/β-catenin pathway. We found β-catenin overexpression couldnot promote DDX39 expression (data not show), DDX39 was an upstream molecule of Wnt/β-catenin pathway, and was a good target for HCC therapy, how DDX39 activates Wnt/β-catenin still is to be explored.

In summary, DDX39 promoted HCC growth and metastasis by activating Wnt/β-catenin pathway, and was a potential target for HCC therapy.

## Materials and methods

### Cell culture

Immortalized normal liver cancer LO_2_ and HCC cells Huh7, SNU-475, SNU-387, Huh1, SK-Hep1, HepG2, and HepB3 were cultured using DMEM supplemented with 10% fetal bovine serum (Hyclone). Cells were maintained in a humidified atmosphere at 37 °C with 5% CO_2_.

### Clinical samples, histology, and immunohistochemistry (IHC)

A cohort of 110 paraffin-embedded specimens was collected during surgical procedures from patients with HCC according to a protocol approved by the institutional review board of the Third Affiliated Hospital of Sun Yat-sen University. Frozen HCC specimens, along with matched normal tissues were available form four patients. All patients provided written, informed consent for participation in the study and provision of tumor samples. IHC assay were carried out as described^27,28^. The antibody used was DDX39 (ab96621, Abcam). Immunohistochemical staining of slides was reviewed and scored by two pathologists independent. The staining index (SI) was calculated as the product of the staining intensity and the proportion of positive cell scores. For histology of mice lung, tissues were collected and fixed with 4% paraformaldehyde overnight and embedded in paraffin, then sectioned at 5 μm. Finally, haematoxylin and eosin stained. The images were captured using the AxioVision Rel.4.6 computerized image analysis system (Carl Zeiss Co Ltd, Jena, Germany).

### qRT-PCR

Total RNA was extracted using Trizol reagent (Life Technologies), and reversely transcribed into cDNA using PrimeScript™ RT reagent Kit (TaKaRa), Oligo dT_18_ was used as the primer. Relative gene expression levels were examined using SYBR® *Premix Ex Taq*™ II (Tli RNaseH Plus) (TaKaRa) on a CFX96 Touch Real-time PCR Detection system (Bio-Rad). GAPDH was used as the internal control.

### Protein isolation and western blot

Total proteins were extracted using RIPA buffer (50 mM Tris (pH 7.4), 1 mM EDTA, 150 mM NaCl, 1% NP-40, 0.5% sodium deoxycholate) supplemental with protease inhibitors (Roche). Western blot assay was performed as previously described^28^ with antibodies against DDX39 (ab96621, Abcam), TCF4 (#2596, Cell Signaling Technology), LEF1 (#2230, Cell Signaling Technology) and GAPDH (G8795, Sigma). For nuclear proteins extraction, KeyGEN Nuclear and Cytoplasmic Protein Extraction Kit (KGP150, KeyGEN BioTECH) was used, the antibodies against β-catenin (#8480, Cell Signaling Technology) and EF-1α (#2551, Cell Signaling Technology) were used.

### Vectors and infection

Full-length human DDX39 cDNA was subcloned into the pMSCV-pur retroviral vector (Clontech) to generate pMSCV-DDX39 vector (indicated as DDX39), the empty vector was used as the negative control (indicated as Vector). Two short hairpin RNAs (shRNAs) oligonucleotides sequences against DDX39 was cloned into the pSuper-retro-pur retroviral vector (OligoEngine) to generate pSuper-DDX39 shRNAs (indicated as shDDX39#1 and shDDX39#2, respectively), The sequences of shRNAs were: shDDX39#1, 5′-GCGAGTCAACATCGTCTTTA-3′ and shDDX39#2, 5′-CCAGGTGATAATCTTCGTCAA-3′. the scramble shRNA sequence was cloned pSuper-pur vector and used as the negative control (indicated as Scramble). pMSCV-DDX39 vector, pMSCV empty vector, pSuper-DDX39 shRNA1, pSuper-DDX39 shRNA2 or pSuper-pur vector were co-transfected with pCL into 293T using Lipofectamine 3000 (Life Technologies). The retroviral supernatants were collected 48 h after transfection and filtered through a 0.45 μm filter. Supernatants plus polybrene (Sigma) were infected with growing HCC cells, after 6 h, the supernatants were replaced by fresh medium. Puromycin (Sigma) was sued to screen stably cell lines. The small interferences RNAs (siRNAs) of TCF4 and LEF and the negative control were purchased from Guangzhou RiboBio Co., Ltd. siRNAs were transfected into HCC cells using Lipofectamine 3000.

### Luciferase reporter assay

The reporter plasmids containing wild-type (CCTTTGATC; TOPflash, plasmid 16558) or mutated (CCTTTGGCC; FOPflash, plasmid 16559) TCF/LEF DNA binding sites were purchased form Addgene, and co-transfected with pRL-TK Renilla plasmid into HCC cells using Lipofectamine 3000 (Life Technologies), respectively. Forty-eight hours after transfection, luciferase activity was analyzed using the Dual-Glo Luciferase Assay Kit (Promega) according to the manufacturer’s protocol. Experiments were performed in triplicates.

### Cell migration and invasion assay

Tumor cell migration and invasion were determined using wound healing, transwell assay and 3D spheroid invasion assay performed as described^29^. For 3D spheroid invasion assay, cells were seeded on 2% Materigel (BD) coated in 24-well plates, and medium was changed every other day. Cells which have formed a 3D spherical structure were photographed for 10 days.

### Animal study

All animal experiments were performed under the protocols approved by the Institutional Animal Care and Use Committee of the Third Affiliated Hospital of Sun Yat-sen University. Four- to 6-week-old nu/nu athymic BALB/cA mice were purchased from the Experimental Animal Center of the Guangzhou University of Chinese Medicine. A total of 5×10^6^ Hep3B with DDX39 overexpression or knockdown were orthotopically injected into the liver parenchyma of mice to observe for lung metastasis (*n* = 6), the mice were fed for 40 days, then were killed, tumors were excised. The activity of aspartate aminotransferase (AST) and alanine aminotransferase (ALT) in blood were examined.

### Statistical analysis

SPSS 19.0 was used to perform all statistical analyses. All data from at least three independent experiments are presented as the mean ± s.d. Comparisons between different groups were analyzed using Student’s *t*-test, survival curves were derived from Kaplan–Meier estimates, multivariate Cox-regression analysis was used to determine the prognostic value of DDX39 levels and other clinicopathologic characteristic. GSEA was performed using http://software.broadinstitute.org/gsea/index.jsp^30^. *p* < 0.05 was considered to be statistically significant.

## Electronic supplementary material

Supplemental Figure 1

Supplemental Figure 2

Supplementary figure legends
