# Supplementary figures and images for: DDX39 promotes hepatocellular carcinoma growth and metastasis through activating Wnt/β-catenin pathway
Source: Cell Death Dis. 2018 Jun 4;9(6):675. doi: 10.1038/s41419-018-0591-0 (PMC5986742; doi:10.1038/s41419-018-0591-0)

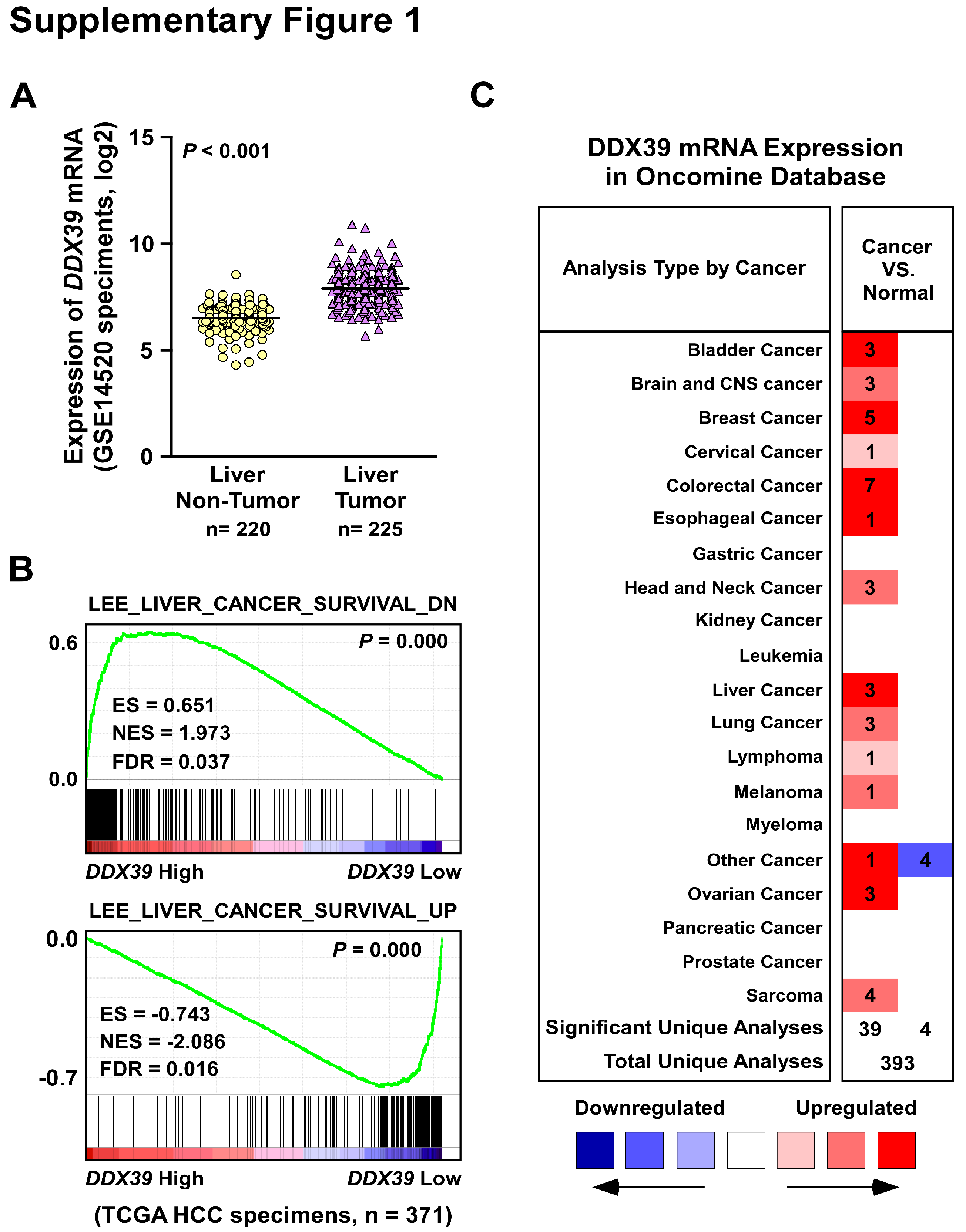

Supplement: Supplementary file 1 — Supplemental Figure 1 [file 41419_2018_591_MOESM1_ESM.tif]

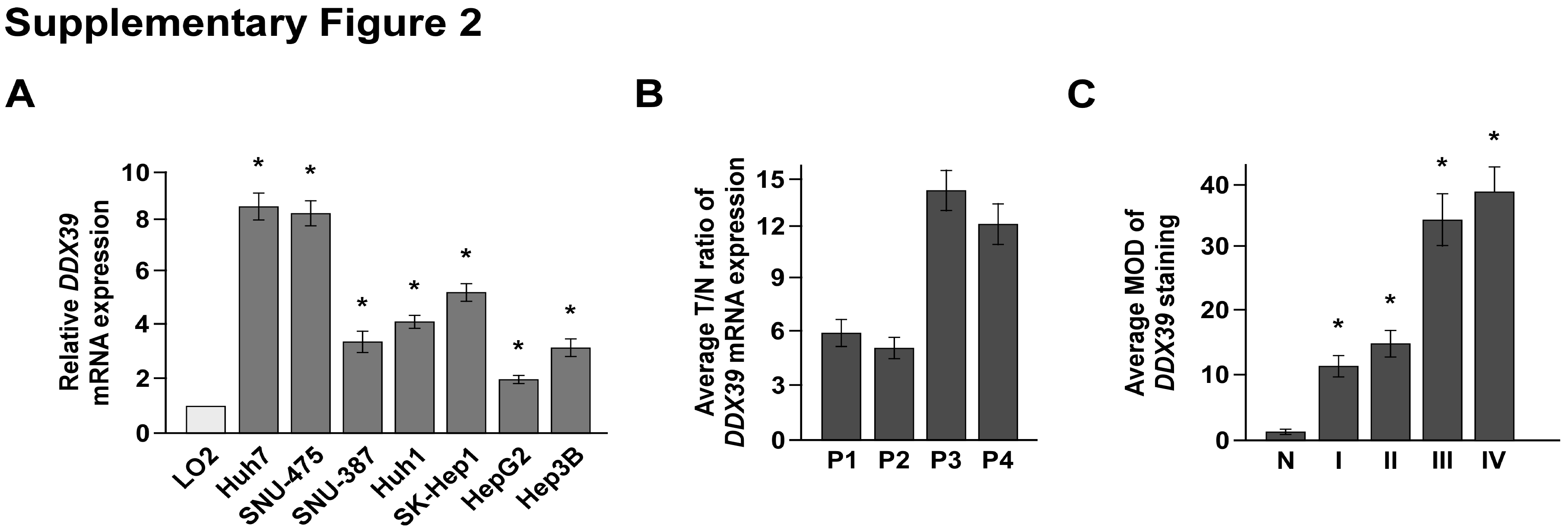

Supplement: Supplementary file 2 — Supplemental Figure 2 [file 41419_2018_591_MOESM2_ESM.tif]
