# Supplementary material for: DDX39 promotes hepatocellular carcinoma growth and metastasis through activating Wnt/β-catenin pathway
Source: Cell Death Dis. 2018 Jun 4;9(6):675. doi: 10.1038/s41419-018-0591-0 (PMC5986742; doi:10.1038/s41419-018-0591-0)
Supplement: Supplementary file 3 — Supplementary figure legends [file 41419_2018_591_MOESM3_ESM.docx]

**Supplemental figure legends**

**Supplemental Figure 1. DDX39 is upregulated in HCC tissues. A.** DDX39 was upregulated in HCC tissues compared to normal liver tissues. Data came from GSE14520. **B.** GSEA assay of the correlation between DDX39 levels and the survival of HCC patient using TCGA data. **C.** DDX39 mRNA expression in various tumors ,data was downloaded from Oncomine database.

**Supplemental Figure 2. DDX39 mRNA levels upregulate in HCC cells and tissues.** **A.** qRT-PCR analyzed the expression of DDX39 in normal liver cell and HCC cells. **B.** qRT-PCR analyzed the expression of DDX39 in four HCC tissues (T) and their matched normal liver tissues (N). **C.** Statistical quantification of the average MODs of DDX39 staining between normal liver tissues and HCC tissues at different clinical stages. Data are shown as means ± SD. *p< 0.05.
